# Supplementary material for: Structural Basis for pH-gating of the K+ channel TWIK1 at the selectivity filter
Source: Nat Commun. 2022 Jun 9;13:3232. doi: 10.1038/s41467-022-30853-z (PMC9184524; doi:10.1038/s41467-022-30853-z)
Supplement: Supplementary file 1 — Supplementary Information [file 41467_2022_30853_MOESM1_ESM.pdf]

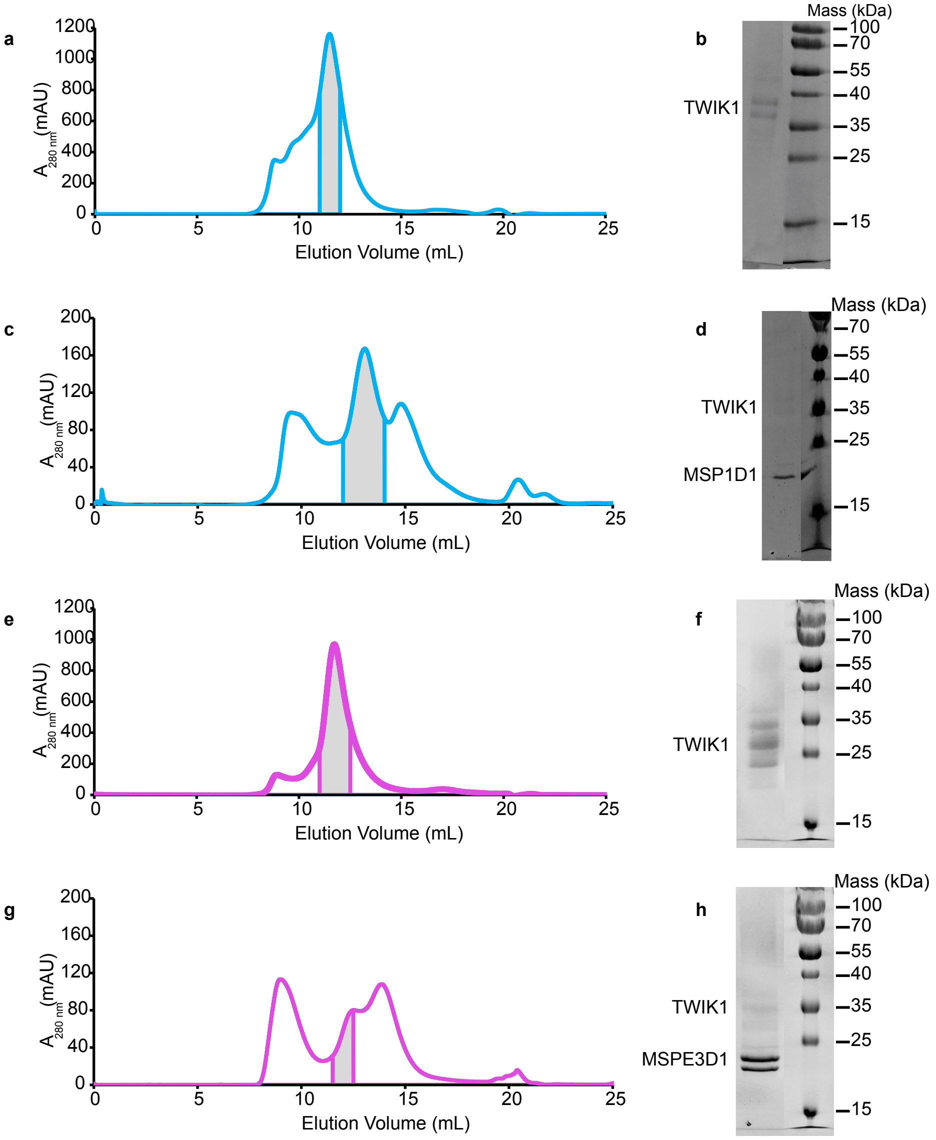

Supplementary Figure 1. Purification and reconstitution of TWIK1.
(a-d) Data for assembly of TWIK1-MSP1D1 nanodisc samples at pH 7.4. **(a)** Chromatogram from a Superdex 200 gel filtration of TWIK1 purified in DDM/CHS detergent. **(b)** Coomassie-stained SDS-PAGE of pooled TWIK1-containing fractions (indicated by gray bar in **(a)**). **(c)** Chromatogram from Superdex 200 gel filtration of TWIK1 reconstituted in MSP1D1 lipid nanodiscs. **(d)** Coomassie-stained SDS-PAGE of final pooled TWIK1-MSP1D1 nanodisc sample (indicated by gray bar in **(c)**). **(e-h)**, Same as **(a-d)**, but for TWIK1-MSPE3D1 nanodisc samples at pH 5.5. Data in **(a-h)** are from the preparation used for structure determination. Source data for **(b,d,f,h)** are provided as a Source Data file.

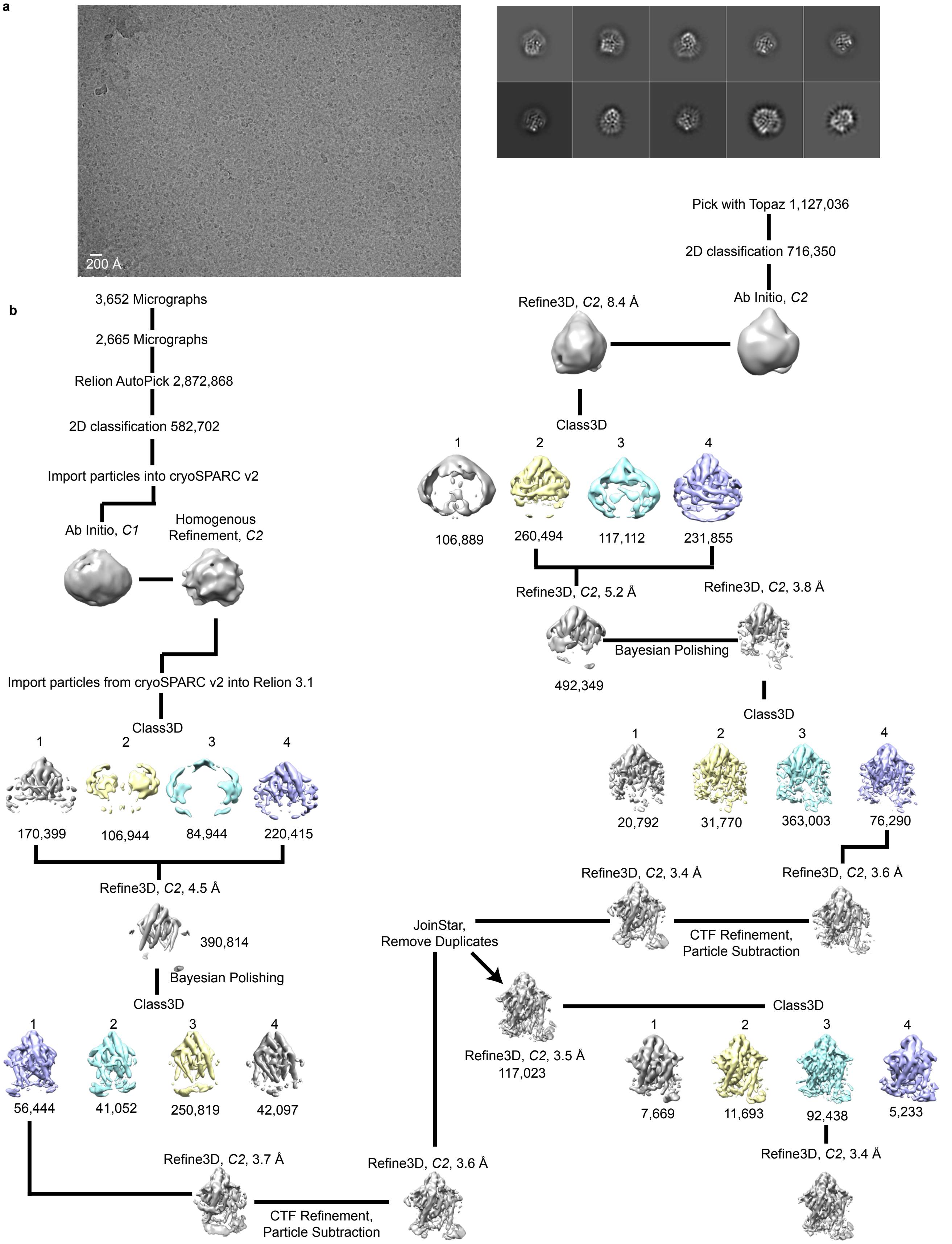

Supplementary Figure 2. Cryo-EM processing pipeline for TWIK1 at pH 7.4.
(a) Example micrograph (left) and selected 2D class averages (right) of TWIK1 in MSP1D1 nanodiscs at pH 7.4. 2D classification was performed in Relion with an extracted box size of 160 pix. **(b)** cryo-EM data processing steps in Relion and cryoSPARC2. See Methods for details.

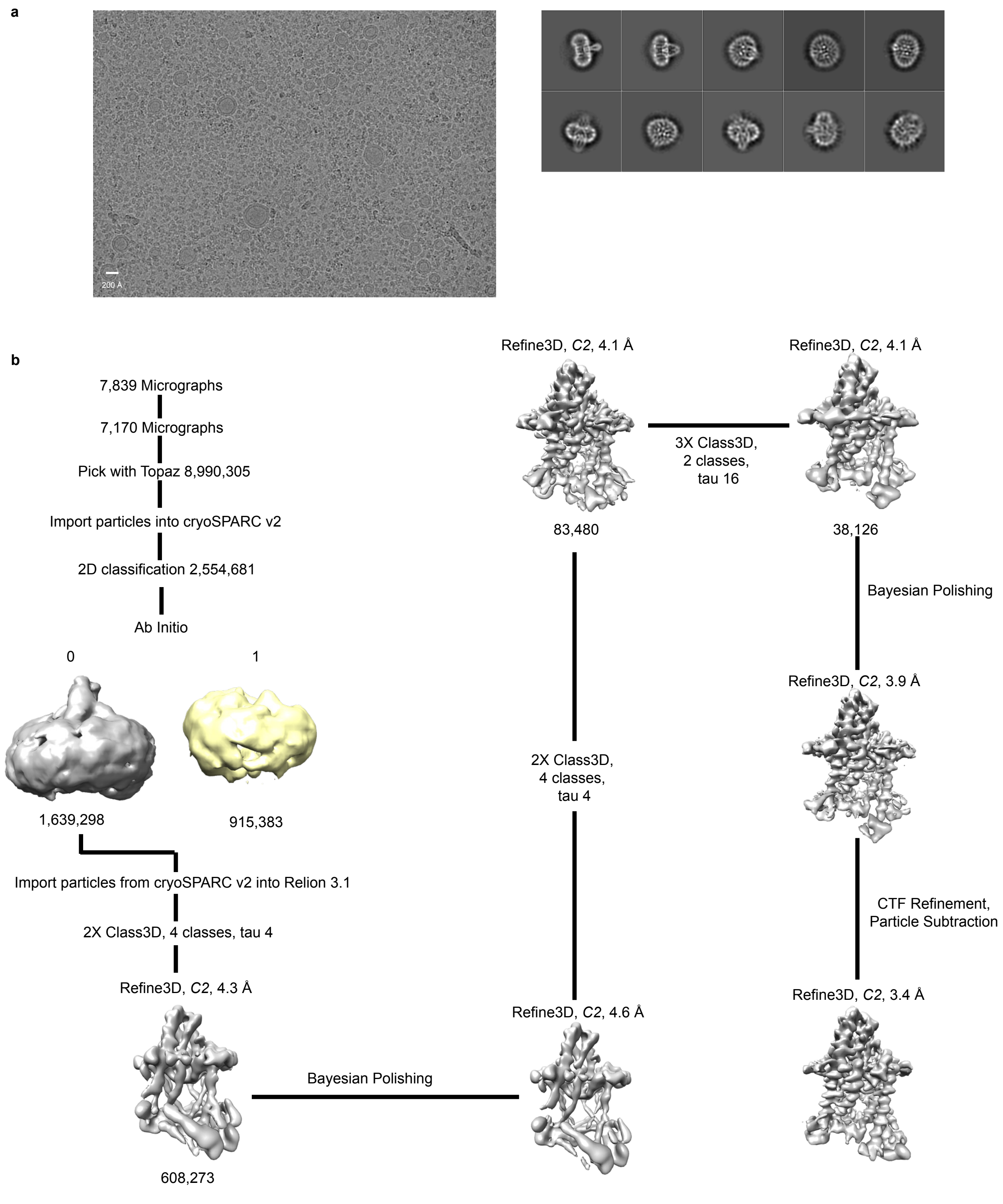

Supplementary Figure 3. Cryo-EM processing pipeline for TWIK1 at pH 5.5.
(a) Example micrograph (left) and selected 2D class averages (right) of TWIK1 in MSPE31D1 nanodiscs at pH 5.5. 2D classification was performed in Relion with an extracted box size of 200 pix. **(b)** cryo-EM data processing steps in Relion and cryoSPARC2. See Methods for details.

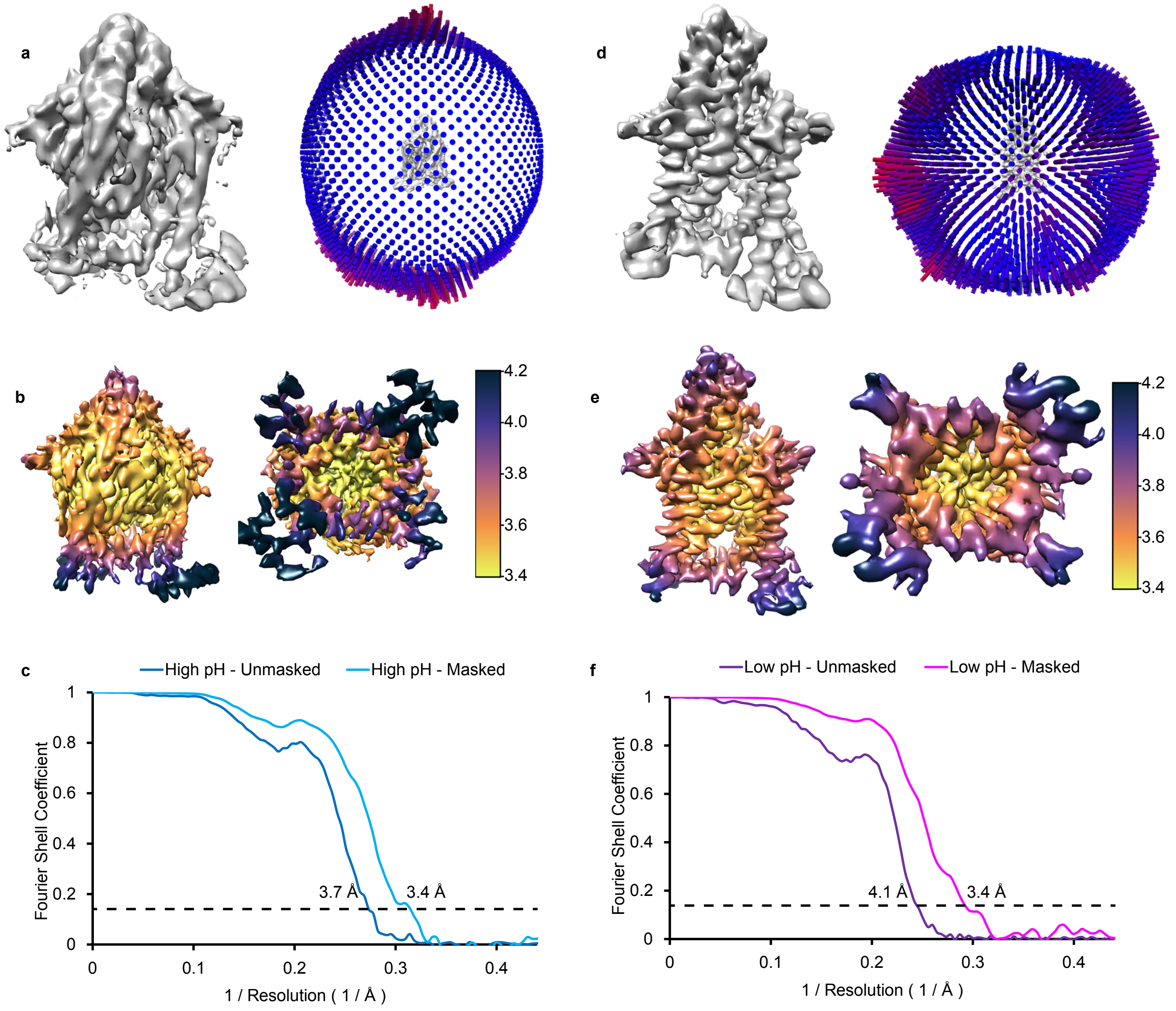

Supplementary Figure 4. Cryo-EM validation.
Validation data for **(a-c)** pH 7.4 and **(d-f)** pH 5.5. **(a,d)** Angular distribution of particles used in final refinement with final map for reference. **(b,e)** Local resolution estimated in Relion colored as indicated on the final map. **(c,f)** Fourier Shell Correlation (FSC) relationships between the two unmasked or masked half-maps from refinement used for calculating overall resolution at 0.143.

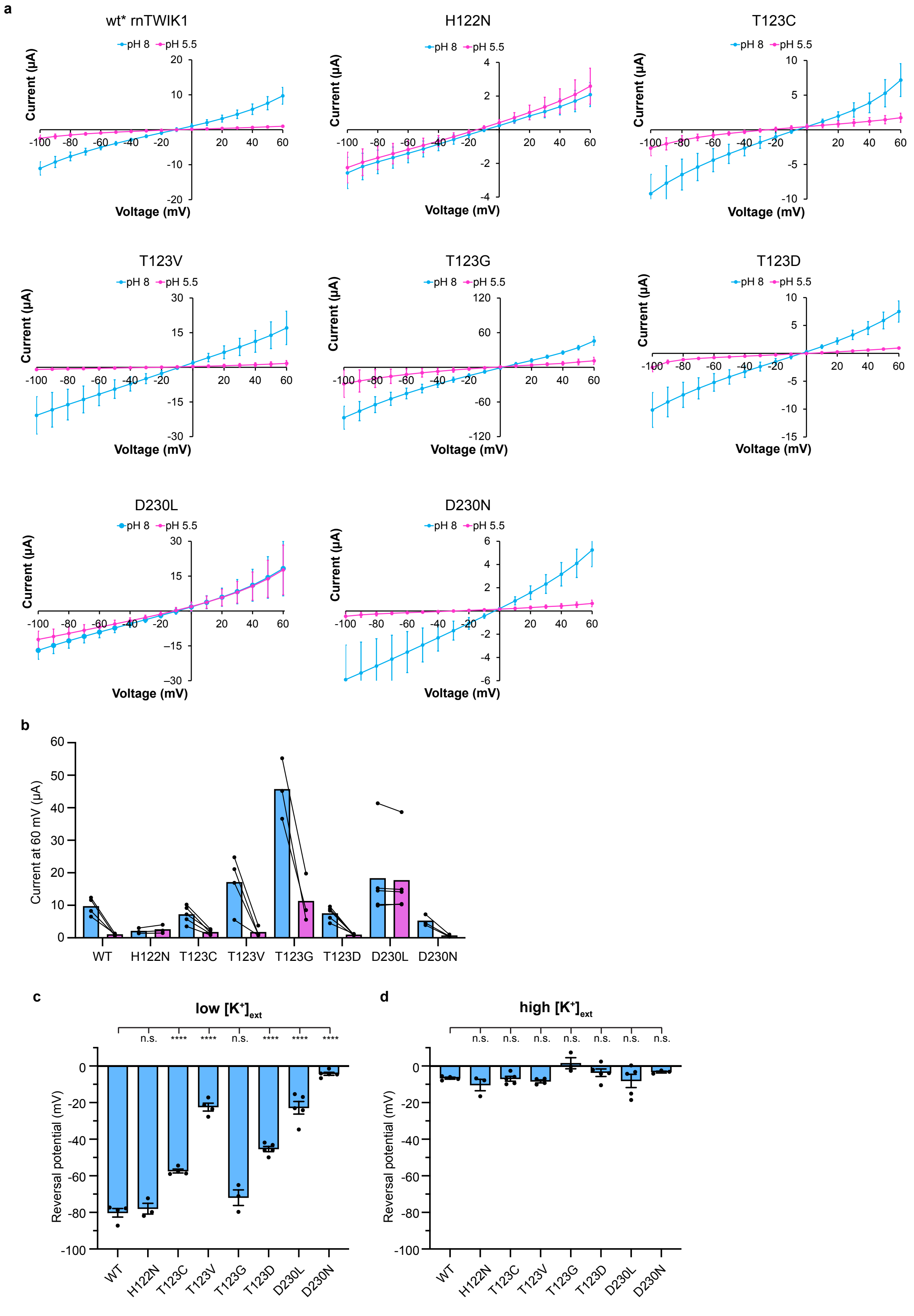

Supplementary Figure 5. Functional characterization of TWIK1 and TWIK1 mutant channels.
(a) Average current-voltage plot recorded in high $[K^+]_{ext}$ for each TWIK1 construct used in this study at pH_{ext} 8 and pH_{ext} 5.5. **(b)** Average current for each mutant at +60 mV and pH_{ext} 8 or 5.5 from **(a).** Lines connect data from a single cell. **(c,d)** Reversal potentials at pH_{ext} 8 and **(c)** low $[K^+]_{ext}$ (2 mM) and **(d)** high $[K^+]_{ext}$ (96 mM): wild-type TWIK1 (-80.2 ± 2.3 and -6.3 ± 0.5), H122N (78.0 ± 2.9 and -10.4 ± 3.1), T123C (-57.4 ± 1.0 and -6.9 ± 1.3), T123V (-22.4 ± 2.2 and -8.4 ± 0.8), T123G (-71.9 ± 4.3 and 1.5 ± 3.0), T123D (-45.4 ± 1.4 and -3.6 ± 2.1), D230L (-22.8 ± 3.4 and -8.1 ± 3.7), and D230N (-4.3 ± 0.8 and -3.2 ± 0.5). Data in **(a-d)** are mean ± sem for n = 4,3,4,4,3,5,5, and 5 cells (low $[K^+]_{ext}$) or n=4,3,5,4,3,5,5, and 3 cells (high $[K^+]_{ext}$). Differences assessed with one-way analysis of variance (ANOVA) with Dunnett correction for multiple comparisons (****P < 0.0001, n.s.: not significant)). Source data for **(a-d)** are provided as a Source Data file.

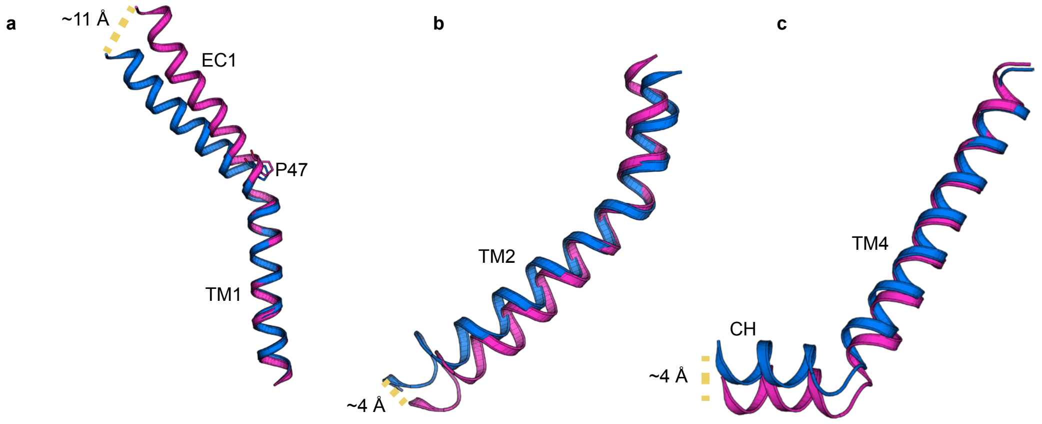

Supplementary Figure 6. Indirect movements of TM1, TM2 and TM4 in response to pH_{ext}.
(a) Overlay of TM1 at a high pH (blue), and low pH (pink). **(b)** Same as in (a) but with TM2. **(c)** Same as in **(a)** and **(b)**, but with TM4. TM1 was aligned using residues 24-46 for each model in **(a)**. The SF and pore helices of each model were used for alignment in **(b)** and **(c)**.

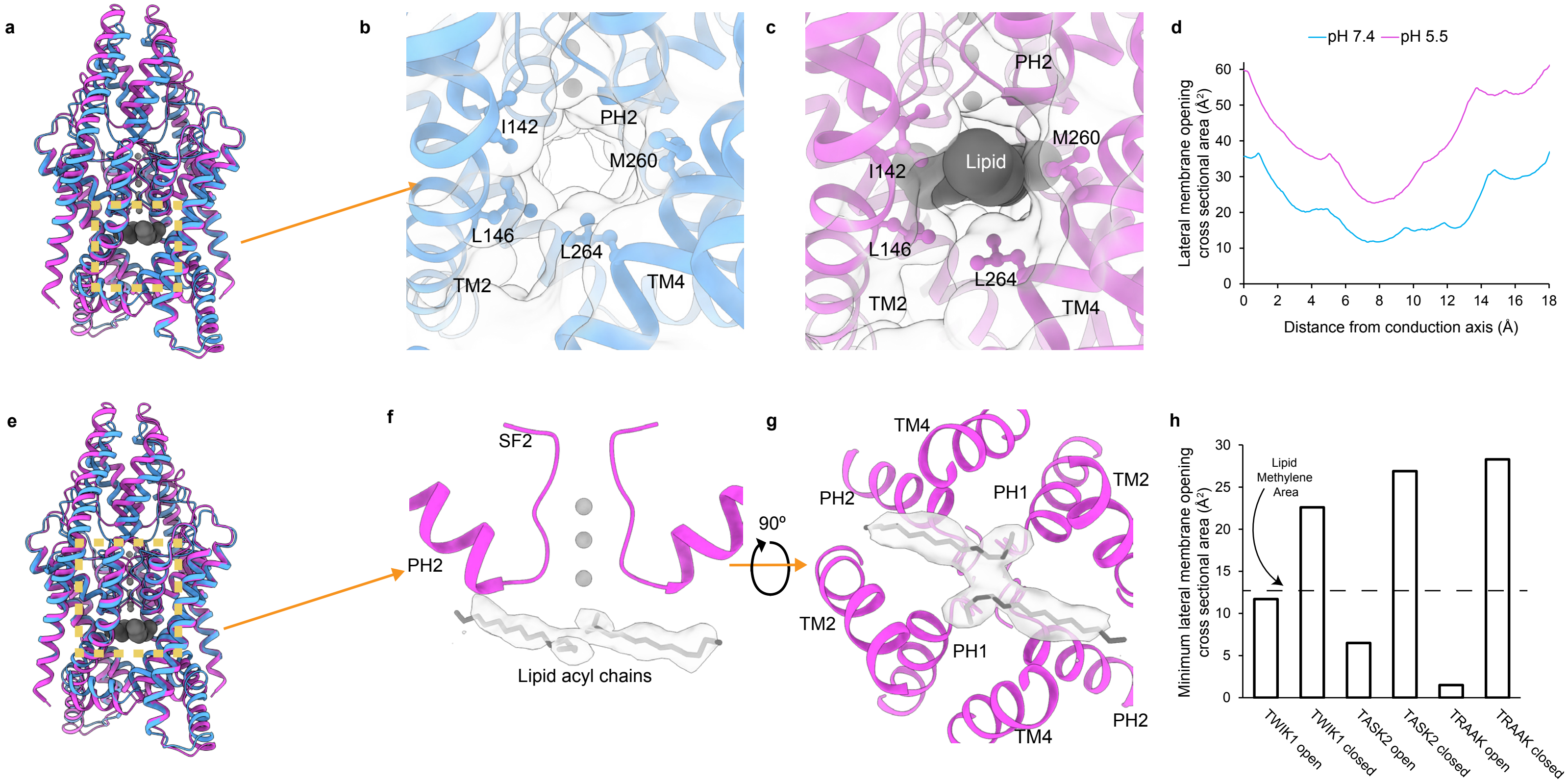

Supplementary Figure 7. Differences in the lateral membrane opening of TWIK1 at a high and low pH.
(a) Overlay of TWIK1 at pH 7.4 (blue) and 5.5 (magenta) viewed from the membrane plane with the lateral membrane opening highlighted. **(b,c)** Zoomed view of the lateral membrane opening at **(b)** pH 7.4 and **(c)** pH 5.5. **(d)** Cross-sectional area of the lateral membrane opening as a function of distance from the conduction axis. **(e)** Overlay of TWIK1 at pH 7.4 (blue) and 5.5 (magenta) viewed from membrane plane with the selectivity filter and cavity highlighted. **(f,g)** Zoomed view of the TWIK1 selectivity filter and cavity at low pH from the **(f)** membrane plane and **(g)** cytoplasm with cryo-EM density for acyl chains shown in gray. **(h)** Comparison of the minimum constriction of the lateral membrane opening in TWIK1, TASK2, and TRAAK structures (TASK2 open PDB:6wm0; TASK2 closed PDB:6wlv (Li et al, 2020); TRAAK open PDB:4wfe; TRAAK closed PDB:4wff (Brohawn et al, 2014)).

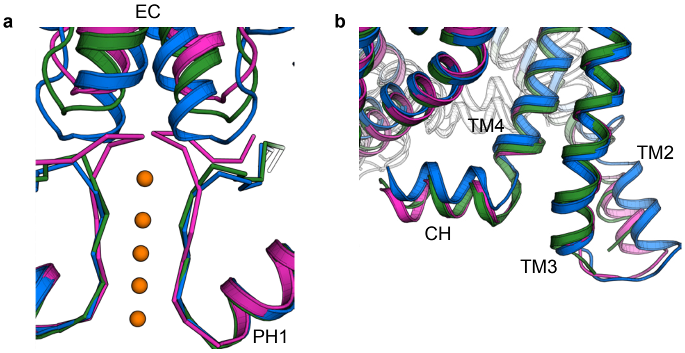

Supplementary Figure 8. Comparison of crystal and cryo-EM structures of TWIK1.
Overlay of TWIK1 in lipid nanodiscs at a high pH (blue), at a low pH (magenta), and in detergent micelles at a high pH (green, PDB: 3ukm) focusing on the SF **(a)**, or on the intracellular ends of TM2, TM3 and TM4 **(b).**

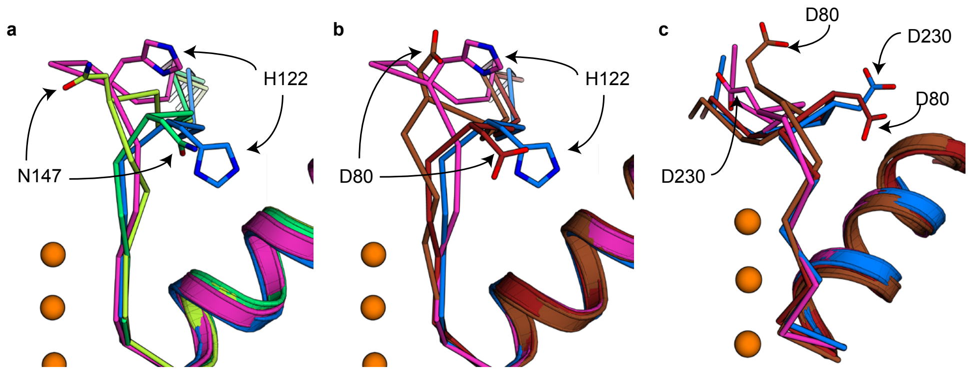

Supplementary Figure 9. Comparison of selectivity filter gates in TWIK1 and other K⁺ channels.
(a) View of SF1 and H122 from the plane of the membrane with TWIK1 at a low pH (magenta) high pH (blue), overlaid with TREK1 at 1 mM K+ (yellow-green, PDB: 6w7c) and TREK1 at 100 mM K+ (lime-green, PDB: 6w83), (Lolicato et al, 2020). **(b)** Same as **(a)**, but with two conformations of KcsA-E71A (brown, PDB: 2atk; dark red, PDB: 1zwi) (Cordero-Morales et al, 2006). **(c)** Same as **(b)**, but comparing SF2 and D230 of TWIK1 with D80 of KcsA-E71A.

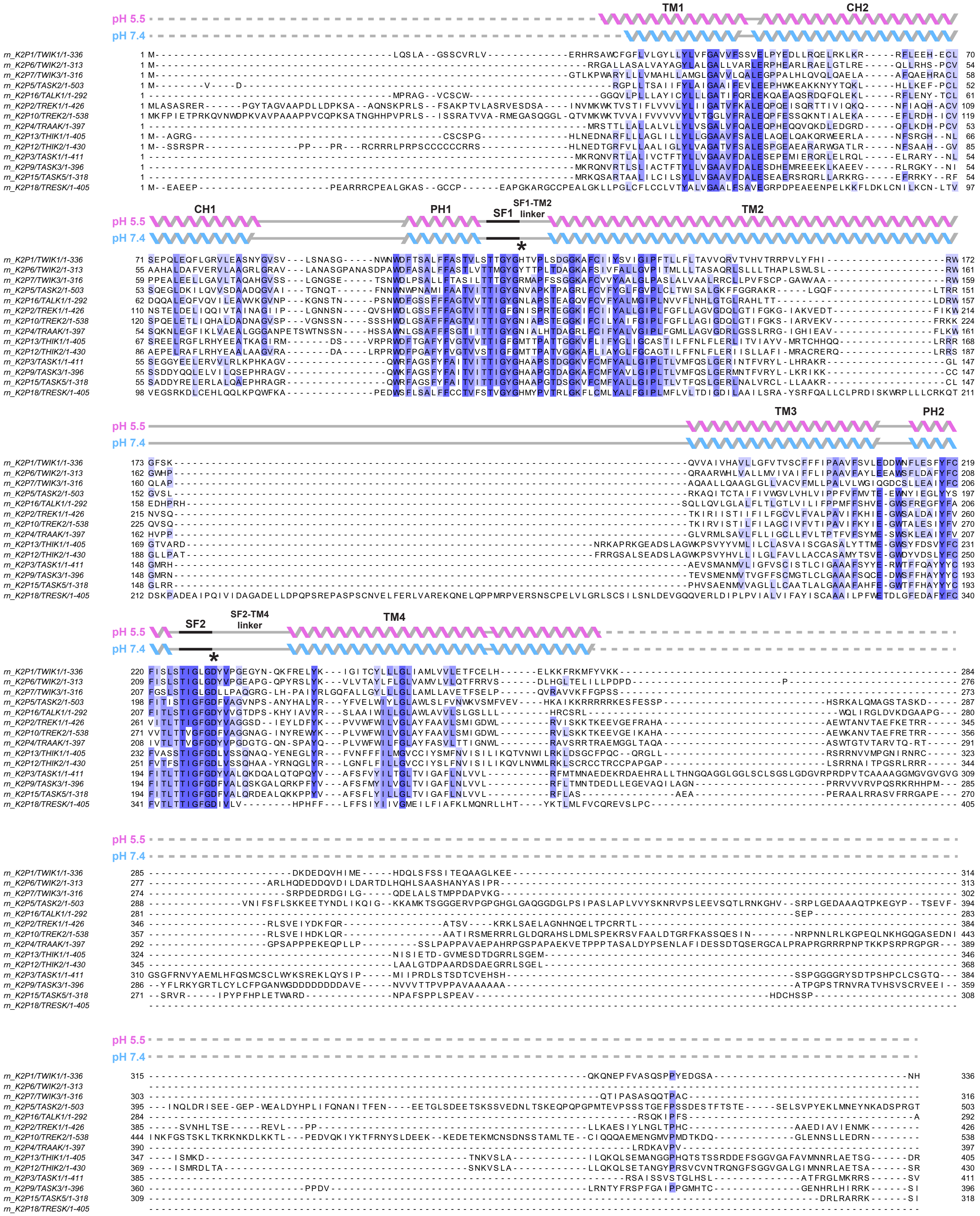

Supplementary Figure 10. Sequence alignment of rat K2P channels.
Alignment of the 14 rat K2P channels colored by conservation in a ramp from white (not conserved) to blue (highly conserved). Secondary structure in pH 5.5 and 7.4 models is shown above the alignment with unmodeled residues indicated by dashed lines. H122 and D230 are highlighted with asterisks.
