# Supplementary material for: Structural Basis for pH-gating of the K+ channel TWIK1 at the selectivity filter
Source: Nat Commun. 2022 Jun 9;13:3232. doi: 10.1038/s41467-022-30853-z (PMC9184524; doi:10.1038/s41467-022-30853-z)
Supplement: Supplementary file 3 — Description of Additional Supplementary Files [file 41467_2022_30853_MOESM3_ESM.pdf]

File name: Supplementary Movie 1

Description: Extracellular view of the TWIK1 selectivity filter morphing from a high pH open conformation to a low pH closed conformation.

File name: Supplementary Movie 2

Description: View from the membrane plane of TWIK1 morphing from a high pH open conformation to a low pH closed conformation
