# Supplementary material for: Structural Basis for pH-gating of the K+ channel TWIK1 at the selectivity filter
Source: Nat Commun. 2022 Jun 9;13:3232. doi: 10.1038/s41467-022-30853-z (PMC9184524; doi:10.1038/s41467-022-30853-z)
Supplement: Supplementary file 6 — Reporting Summary [file 41467_2022_30853_MOESM6_ESM.pdf]

nature portfolio

Corresponding author(s): Brohawn, S.G.

Last updated by author(s): May 10, 2022

Reporting Summary

Nature Portfolio wishes to improve the reproducibility of the work that we publish. This form provides structure for consistency and transparency in reporting. For further information on Nature Portfolio policies, see our Editorial Policies and the Editorial Policy Checklist.

Statistics

For all statistical analyses, confirm that the following items are present in the figure legend, table legend, main text, or Methods section.

n/a	Confirmed	
☐	☒	The exact sample size (*n*) for each experimental group/condition, given as a discrete number and unit of measurement
☐	☒	A statement on whether measurements were taken from distinct samples or whether the same sample was measured repeatedly
☐	☒	The statistical test(s) used AND whether they are one- or two-sided<br>*Only common tests should be described solely by name; describe more complex techniques in the Methods section.*
☒	☐	A description of all covariates tested
☐	☒	A description of any assumptions or corrections, such as tests of normality and adjustment for multiple comparisons
☐	☒	A full description of the statistical parameters including central tendency (e.g. means) or other basic estimates (e.g. regression coefficient) AND variation (e.g. standard deviation) or associated estimates of uncertainty (e.g. confidence intervals)
☐	☒	For null hypothesis testing, the test statistic (e.g. *F*, *t*, *r*) with confidence intervals, effect sizes, degrees of freedom and *P* value noted<br>*Give P values as exact values whenever suitable.*
☒	☐	For Bayesian analysis, information on the choice of priors and Markov chain Monte Carlo settings
☒	☐	For hierarchical and complex designs, identification of the appropriate level for tests and full reporting of outcomes
☒	☐	Estimates of effect sizes (e.g. Cohen's *d*, Pearson's *r*), indicating how they were calculated

Our web collection on statistics for biologists contains articles on many of the points above.

Software and code

Policy information about availability of computer code

Data collection	Sutter Patch v2.2, ChromLab 6.0, SerialEM 3.6
Data analysis	Relion 3.1, MotionCor2, Ctffind 4.1, Cryosparc 2, UCSF PyEM 0.5, Phenix 1.19, Coot 0.9, Molprobity 4.5, PyMOL 2.3.2, ChimeraX 0.6, Prism 8, Topaz 0.2.5, HOLE implemented in Coot 0.9, AlphaFold2

For manuscripts utilizing custom algorithms or software that are central to the research but not yet described in published literature, software must be made available to editors and reviewers. We strongly encourage code deposition in a community repository (e.g. GitHub). See the Nature Portfolio guidelines for submitting code & software for further information.

Data

Policy information about availability of data

All manuscripts must include a data availability statement. This statement should provide the following information, where applicable:

- Accession codes, unique identifiers, or web links for publicly available datasets
- A description of any restrictions on data availability
- For clinical datasets or third party data, please ensure that the statement adheres to our policy

The final cryo-EM maps of TWIK1 in MSP1D1 nanodiscs at pH 7.4 and in MSPE3D1 nanodiscs at pH 5.5 generated in this study have been deposited in the Electron Microscopy Data Bank under accession codes 25168 (https://www.emdataresource.org/EMD-25168) and 25169 (https://www.emdataresource.org/EMD-25169). The final atomic coordinates have been deposited in the PDB under IDs 7SK0 (https://www.rcsb.org/structure/7SK0) and 7SK1(https://www.rcsb.org/structure/7SK1. The original micrograph movies have been deposited in EMPIAR.

Field-specific reporting

Please select the one below that is the best fit for your research. If you are not sure, read the appropriate sections before making your selection.

☒ Life sciences ☐ Behavioural & social sciences ☐ Ecological, evolutionary & environmental sciences

For a reference copy of the document with all sections, see nature.com/documents/nr-reporting-summary-flat.pdf

Life sciences study design

All studies must disclose on these points even when the disclosure is negative.

Sample size	No statistical tests were used to predetermine sample sizes. Sample sizes for electrophysiology were chosen based on established practice in the field and were sufficient as differences between groups were reproducible. See e.g. Li et al. Nature 2020.
Data exclusions	No data were excluded from analyses.
Replication	All attempts at replication were successful. Number of replicates are indicated in legends.
Randomization	Not applicable to our study. No predetermined control and sample groups were used.
Blinding	Not applicable to our study. No populations were preassigned to groups so blinding was not relevant.

Reporting for specific materials, systems and methods

We require information from authors about some types of materials, experimental systems and methods used in many studies. Here, indicate whether each material, system or method listed is relevant to your study. If you are not sure if a list item applies to your research, read the appropriate section before selecting a response.

Materials & experimental systems

n/a	Involved in the study	
☐	☒	Antibodies
☐	☒	Eukaryotic cell lines
☒	☐	Palaeontology and archaeology
☒	☐	Animals and other organisms
☒	☐	Human research participants
☒	☐	Clinical data
☒	☐	Dual use research of concern

Methods

n/a	Involved in the study	
☒	☐	ChIP-seq
☒	☐	Flow cytometry
☒	☐	MRI-based neuroimaging

Antibodies

Antibodies used	Anti-GFP nanobody (Addgene 49172 https://www.addgene.org/49172/)
Validation	Expression plasmid was sequence verified. No other validation was performed as nanobody was successfully used for protein purification (Supplementary Fig. 1).

Eukaryotic cell lines

Policy information about cell lines

Cell line source(s)	Pichia pastoris SMD1163 (Invitrogen)
Authentication	We did not further authenticate the cell lines.
Mycoplasma contamination	We did not test for mycoplasma.
Commonly misidentified lines (See ICLAC register)	No commonly misidentified lines were used in our study.
